# Supplementary material for: Learning improves decoding of odor identity with phase-referenced oscillations in the olfactory bulb
Source: eLife. 2020 Jan 28;9:e52583. doi: 10.7554/eLife.52583 (PMC6986879; doi:10.7554/eLife.52583)
Supplement: Supplementary file 1. [file elife-52583-supp1.docx]

**Table S1. Odorant pairs, implant device, mouse genotype and electrode locations for each experiment**

| **Experiment** | **Implant** | **Coordinates** | **Genotype** | **Odorant pair** | **Odorant name** | **Odorant dilution (v/v)** | **Number of mice** |
| --- | --- | --- | --- | --- | --- | --- | --- |
| Exp1 | Optetrodes | +4.25 mm AP, +0.4 mm ML, -0.53 mm DV | DBH-Cre eNpHR3.0 (see Methods) | APEBexp1 | Acetophenone | 1% | 14 |
|  |  |  |  |  | Ethyl benzoate | 1% |  |
|  |  |  |  | EAPAexp1 | Ethyl acetate (EA) | 0.1% | 11 |
|  |  |  |  |  | EA/Propyl acetate (PA) | 0.05% EA + 0.05% PA |  |
|  |  |  |  | IAMOexp1 | Isoamyl acetate | 1% | 9 |
|  |  |  |  |  | Mineral oil | 100% |  |
| Exp2 | Tetrodes | +4.28 mm AP, +0.05 mm ML, -1.0 mm DV | C57BL/6 | EAPAexp2 | Ethyl acetate | 1% | 9 |
|  |  |  |  |  | Propyl acetate | 1% |  |
|  |  |  |  | IAAPexp2 | Isoamyl acetate | 1% | 6 |
|  |  |  |  |  | Acetophenone | 1% |  |
|  |  |  |  | IAMOexp2 | Isoamyl acetate | 1% | 4 |
|  |  |  |  |  | Mineral oil | 100% |  |

Odorant identities for each pair are listed along with concentrations represented as volume/volume dilutions in mineral oil (MO). In the case of EAPAexp1, EA is 0.1% ethyl acetate and PA is a mixture of 0.05% ethyl acetate + 0.05% propyl acetate. The two recording locations (Exp1 and Exp2) are listed in millimeters with respect to bregma. Note: Cre expression can affect physiological parameters and therefore, the differences found between experiments in this publication can be due to Cre expression (Harno et al., 2013). However, it is not evident to us which of the methodological differences between experiments contributes to statistical differences in measured parameters.


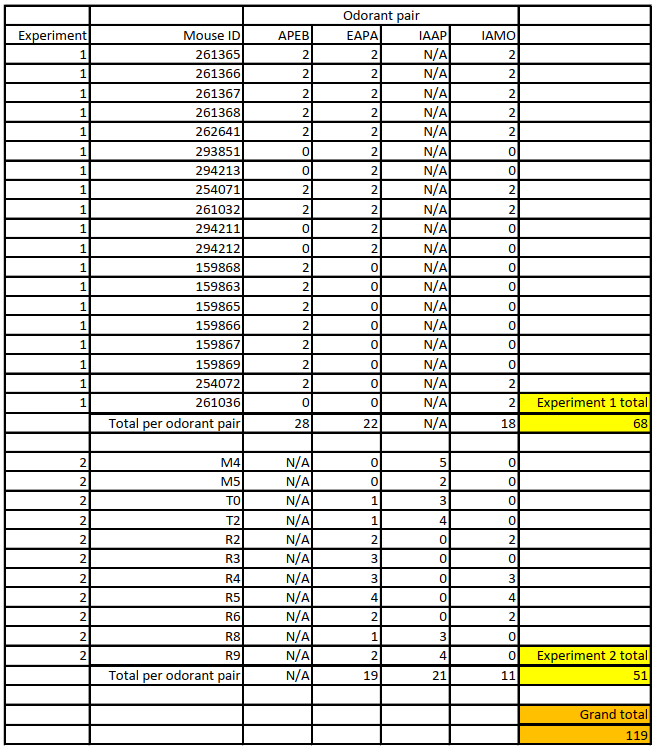


**Table S2. Total sessions per mouse, experiment, and odorant pair.**

Odorant pair abbreviations derived from Table S1. The number of sessions are in the “odorant pair” columns. Each row represents one animal.
