# Supplementary material for: Learning improves decoding of odor identity with phase-referenced oscillations in the olfactory bulb
Source: eLife. 2020 Jan 28;9:e52583. doi: 10.7554/eLife.52583 (PMC6986879; doi:10.7554/eLife.52583)
Supplement: Transparent reporting form [file elife-52583-transrepform.pdf]

eLIFE
1st Floor
24 Hills Road
Cambridge CB2 1JP, UK
P 01223 855340
W elifesciences.org
T @elife

eLife's transparent reporting form

We encourage authors to provide detailed information *within their submission* to facilitate the interpretation and replication of experiments. Authors can upload supporting documentation to indicate the use of appropriate reporting guidelines for health-related research (see EQUATOR Network), life science research (see the BioSharing Information Resource), or the ARRIVE guidelines for reporting work involving animal research. Where applicable, authors should refer to any relevant reporting standards documents in this form.

If you have any questions, please consult our Journal Policies and/or contact us: editorial@elifesciences.org.

Sample-size estimation

- You should state whether an appropriate sample size was computed when the study was being designed
- You should state the statistical method of sample size computation and any required assumptions
- If no explicit power analysis was used, you should describe how you decided what sample (replicate) size (number) to use

Please outline where this information can be found within the submission (e.g., sections or figure legends), or explain why this information doesn't apply to your submission:

We did not perform a power analysis because when we started the study we did not know the magnitude of the changes in PAC parameters that would take place when the animal learns to discriminate the odorants. We used the same number of mice as in past studies with tetrodes and electrodes in the Restrepo laboratory. In addition, we performed the analysis on two experimental data sets that were obtained in separate experiments by the two first authors. The analysis yielded significant results, estimated using the generalized linear model, for both data sets. Furthermore, we used complementary bootstrap 95% confidence interval (CI) calculations that have been recommended for their use in complement to p values. CI quantifies the uncertainty of the measure by presenting the range within which the true effect size is likely to lie (Halsey et al. Nature Methods 12:179-185, 2015, see also Levine et al., Pharmacotherapy 21:405–409, 2001).

Replicates

- You should report how often each experiment was performed
- You should include a definition of biological versus technical replication
- The data obtained should be provided and sufficient information should be provided to indicate the number of independent biological and/or technical replicates
- If you encountered any outliers, you should describe how these were handled
- Criteria for exclusion/inclusion of data should be clearly stated
- High-throughput sequence data should be uploaded before submission, with a private link for reviewers provided (these are available from both GEO and ArrayExpress)

eLife Sciences Publications, Ltd is a limited liability non-profit non-stock corporation incorporated in the State of Delaware, USA, with company number 5030732, and is registered in the UK with company number FC030576 and branch number BR015634 at the address 1st Floor, 24 Hills Road, Cambridge CB2 1JP | August 2014

Please outline where this information can be found within the submission (e.g., sections or figure legends), or explain why this information doesn't apply to your submission:

We state how many mice we used per odorant pair in Table S1, and we state how many experimental sessions were run for each odorant pair and each mouse in Table S2. We did not exclude outliers.

Statistical reporting

- Statistical analysis methods should be described and justified
- Raw data should be presented in figures whenever informative to do so (typically when N per group is less than 10)
- For each experiment, you should identify the statistical tests used, exact values of N, definitions of center, methods of multiple test correction, and dispersion and precision measures (e.g., mean, median, SD, SEM, confidence intervals; and, for the major substantive results, a measure of effect size (e.g., Pearson's r, Cohen's d)
- Report exact p-values wherever possible alongside the summary statistics and 95% confidence intervals. These should be reported for all key questions and not only when the p-value is less than 0.05.

Please outline where this information can be found within the submission (e.g., sections or figure legends), or explain why this information doesn't apply to your submission:

Yes, we followed all bullet points listed above. The statistical methods are detailed in the methods and p values are stated in the results and figure legends. Individual data points and CIs are shown when appropriate. We reported p<0.05 and p<0.001, but we would be glad to report exact values.

(For large datasets, or papers with a very large number of statistical tests, you may upload a single table file with tests, Ns, etc., with reference to sections in the manuscript.)

Group allocation

- Indicate how samples were allocated into experimental groups (in the case of clinical studies, please specify allocation to treatment method); if randomization was used, please also state if restricted randomization was applied
- Indicate if masking was used during group allocation, data collection and/or data analysis

Please outline where this information can be found within the submission (e.g., sections or figure legends), or explain why this information doesn't apply to your submission:

We do not have group allocation in this study. All mice were trained to discriminate several pairs of odorants as detailed in the methods. The comparisons were: 1)Between sets of trials recorded when the animal was naïve (defined as <65 percent correct in discrimination in the go-no go task) vs. proficient (>=80%). 2)Between trials for the rewarded vs the unrewarded odorant. 3) Between the peak and trough of theta phase for the computation of the phase-referenced power. Outliers were not excluded.

Additional data files ("source data")

- We encourage you to upload relevant additional data files, such as numerical data that are represented as a graph in a figure, or as a summary table
- Where provided, these should be in the most useful format, and they can be uploaded as "Source data" files linked to a main figure or table
- Include model definition files including the full list of parameters used
- Include code used for data analysis (e.g., R, MatLab)
- Avoid stating that data files are "available upon request"

Please indicate the figures or tables for which source data files have been provided:

eLIFE
1st Floor
24 Hills Road
Cambridge CB2 1JP, UK
P 01223 855340
W elifesciences.org
T @elife

The Matlab code used for data analysis has been deposited to https://github.com/restrepd/drgMaster. Upon publication we will deposit the data in an open source repository (either GigaDB: http://gigadb.org or Figshare: https://figshare.com/features).
